# Supplementary figures and images for: Longitudinal and regional association between dietary factors and prevalence of Crohn’s disease in Japan
Source: PLoS One. 2024 May 22;19(5):e0300580. doi: 10.1371/journal.pone.0300580 (PMC11111081; doi:10.1371/journal.pone.0300580)

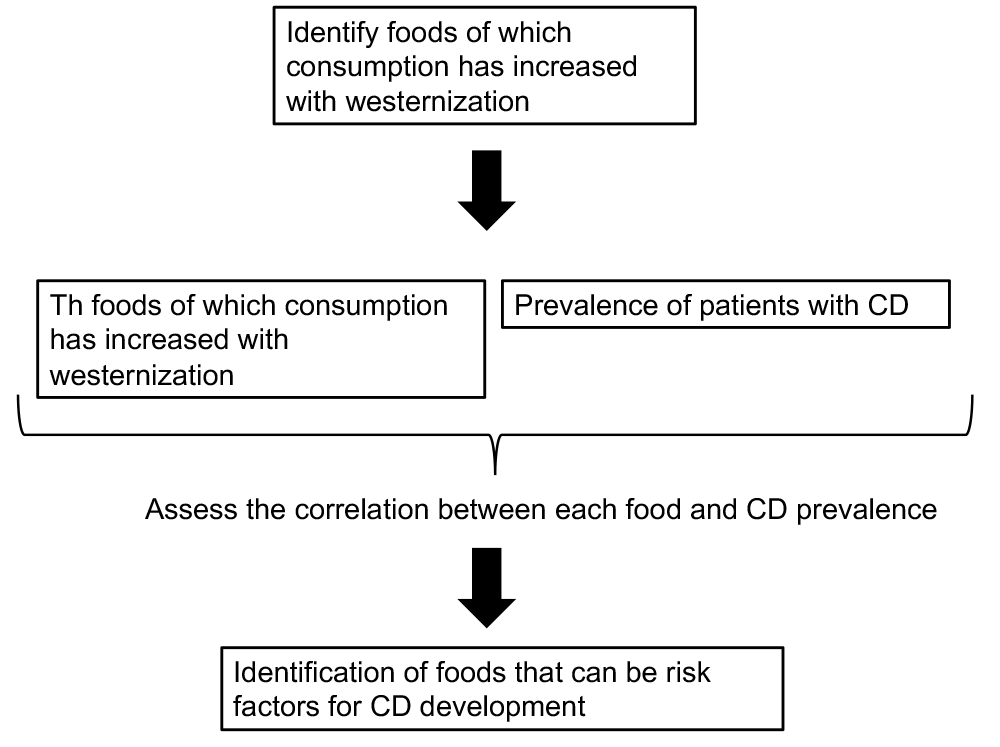

Supplement: S1 Fig — The study design of this research. (TIF) [file pone.0300580.s001.tif]
