# Supplementary material for: Longitudinal and regional association between dietary factors and prevalence of Crohn’s disease in Japan
Source: PLoS One. 2024 May 22;19(5):e0300580. doi: 10.1371/journal.pone.0300580 (PMC11111081; doi:10.1371/journal.pone.0300580)
Supplement: Attachment — Submitted filename: revision.pdf [file pone.0300580.s005.pdf]

General comment:

The topic approached by authors of this paper is interesting and overall, I believe the study has relevant information hereby suitable for publication after revision.

The authors clearly explained their work throughout the manuscript. I just have some remarks/suggestions to point out.

The study found a correlation between increased meat consumption and CD prevalence in Japan, as well as reduced intake of fruits and vegetables. Moreover, it found a peak in meat consumption between the ages of 15-19 years old for both males and females in Japan, as well as a decrease in the consumption of fruits and vegetables in individuals aged between 20-30 and 20 years, respectively.

Specific comments:

In Abstract:

- The results should be reported in a statistical and accurate manner. (for example: increase in meat consumption: which percent or value? The P-value?)

In Methods:

- Meat consumption: the author mean just red meat or is poultry meat included?

In Results & Discussion:

- Fig 3: The units in the horizontal and vertical axes are not specified. Therefore, this figure cannot be understood on its own.
- The study relied on self-reported data, which may be subject to recall bias and other inaccuracies.
- The study did not take into account other potential environmental factors such as smoking, air pollution, and other lifestyle factors.
