# Supplementary material for: Longitudinal and regional association between dietary factors and prevalence of Crohn’s disease in Japan
Source: PLoS One. 2024 May 22;19(5):e0300580. doi: 10.1371/journal.pone.0300580 (PMC11111081; doi:10.1371/journal.pone.0300580)
Supplement: Attachment — Submitted filename: review plose one.docx [file pone.0300580.s004.docx]

Dear authors,

Your study is a valuable research with an interesting topic that aims to investigate the longitudinal and regional association between dietary factors and prevalence of Crohn’s disease in Japan. To improve the study, it is better to consider the following comments:

1- Make the introduction shorter.
2- Mention a statistic of Crohn's disease in Japan in the introduction.
3- The design of study is not mentioned.
4- Write about the ecological fallacy of your research in the limitation section.
5- I did not see the regression table in the results.
6- The effect measure for correlations should be written in the results.
