# Supplementary material for: Longitudinal and regional association between dietary factors and prevalence of Crohn’s disease in Japan
Source: PLoS One. 2024 May 22;19(5):e0300580. doi: 10.1371/journal.pone.0300580 (PMC11111081; doi:10.1371/journal.pone.0300580)
Supplement: Attachment — Submitted filename: Response to Reviewers.docx [file pone.0300580.s006.docx]

We agree with you and have incorporated this suggestion throughout our paper. As instructed, we have attempted to succinctly explain changes made in reaction to all comments. We reply to each comment in point-by-point fashion.

Editor suggestions

1. Please ensure that your manuscript meets PLOS ONE's style requirements, including those for file naming.

●Response: We checked templates and modified the style as indicated.

(p2 line29; p4 line65, p8 line124,125,131,134; p10 line177; p11 line184; p15 line 245; p19 line311, 320; p20 line326)

2. We note that your Data Availability Statement is currently as follows: “All relevant data are within the manuscript and its Supporting Information files.” Please confirm at this time whether or not your submission contains all raw data required to replicate the results of your study. Authors must share the “minimal data set” for their submission. PLOS defines the minimal data set to consist of the data required to replicate all study findings reported in the article, as well as related metadata and methods (https://journals.plos.org/plosone/s/data-availability#loc-minimal-data-set-definition). For example, authors should submit the following data: - The values behind the means, standard deviations and other measures reported; - The values used to build graphs; - The points extracted from images for analysis. Authors do not need to submit their entire data set if only a portion of the data was used in the reported study. If your submission does not contain these data, please either upload them as Supporting Information files or deposit them to a stable, public repository and provide us with the relevant URLs, DOIs, or accession numbers. For a list of recommended repositories, please see https://journals.plos.org/plosone/s/recommended-repositories. If there are ethical or legal restrictions on sharing a de-identified data set, please explain them in detail (e.g., data contain potentially sensitive information, data are owned by a third-party organization, etc.) and who has imposed them (e.g., an ethics committee). Please also provide contact information for a data access committee, ethics committee, or other institutional body to which data requests may be sent. If data are owned by a third party, please indicate how others may request data access.

●Response: We have added sets of the data relating to this article as supporting information files (S1 table and S2 table).

3. We note that Figures 2 (I, J) in your submission contain copyrighted images. All PLOS content is published under the Creative Commons Attribution License (CC BY 4.0), which means that the manuscript, images, and Supporting Information files will be freely available online, and any third party is permitted to access, download, copy, distribute, and use these materials in any way, even commercially, with proper attribution. For more information, see our copyright guidelines: http://journals.plos.org/plosone/s/licenses-and-copyright. We require you to either (1) present written permission from the copyright holder to publish these figures specifically under the CC BY 4.0 license, or (2) remove the figures from your submission: a. You may seek permission from the original copyright holder of Figures 2 (I, J) to publish the content specifically under the CC BY 4.0 license. We recommend that you contact the original copyright holder with the Content Permission Form (http://journals.plos.org/plosone/s/file?id=7c09/content-permission-form.pdf) and the following text: “I request permission for the open-access journal PLOS ONE to publish XXX under the Creative Commons Attribution License (CCAL) CC BY 4.0 (http://creativecommons.org/licenses/by/4.0/). Please be aware that this license allows unrestricted use and distribution, even commercially, by third parties. Please reply and provide explicit written permission to publish XXX under a CC BY license and complete the attached form.” Please upload the completed Content Permission Form or other proof of granted permissions as an "Other" file with your submission. In the figure caption of the copyrighted figure, please include the following text: “Reprinted from [ref] under a CC BY license, with permission from [name of publisher], original copyright [original copyright year].” b. If you are unable to obtain permission from the original copyright holder to publish these figures under the CC BY 4.0 license or if the copyright holder’s requirements are incompatible with the CC BY 4.0 license, please either i) remove the figure or ii) supply a replacement figure that complies with the CC BY 4.0 license. Please check copyright information on all replacement figures and update the figure caption with source information. If applicable, please specify in the figure caption text when a figure is similar but not identical to the original image and is therefore for illustrative purposes only.

●Response: We used statistical software “Tableau” to create the figure 2(I, J). There are free and paid versions of "Tableau". We used a free version and we think that’s because these figures became copyrighted images. The data and figures were our original so we re-created these figures using the software, ”Python”.

4. Please review your reference list to ensure that it is complete and correct. If you have cited papers that have been retracted, please include the rationale for doing so in the manuscript text, or remove these references and replace them with relevant current references. Any changes to the reference list should be mentioned in the rebuttal letter that accompanies your revised manuscript. If you need to cite a retracted article, indicate the article’s retracted status in the References list and also include a citation and full reference for the retraction notice.

●Response: We reviewed references and confirmed that it is complete and correct. There is no retracted article in our references.

Reviewer #1 comments

1- Make the introduction shorter.

●Response: We removed several sentences in the introduction.

(p4, line72,73; p4,5 line95-101; p6 line110)

2- Mention a statistic of Crohn's disease in Japan in the introduction.

●Response: We have added a statistic of Crohn’s disease in Japan in the introduction.

(p6 line106,107)

3- The design of study is not mentioned.

●Response: We created the figure (S1 Fig) about the design of study and mentioned about it in the first paragraph of materials and methods section.

(p8 line130)

4- Write about the ecological fallacy of your research in the limitation section.

●Response: We added about the ecological fallacy of my research in the limitation section.

(p18,19 line306-309)

5- I did not see the regression table in the results.

●Response: We created the regression table in the results.

(Table 1.)

6- The effect measure for correlations should be written in the results.

●Response: We added the effect measure (Standardized regression coefficient) in the results.

(p12 line204,212; p13 line215)

7- please provide ethical code of your research in Method section.

●Response: We used only the existing public data, therefore we did not have ethical code for our research.

Reviewer #2 comments

In Abstract:

- The results should be reported in a statistical and accurate manner. (for example: increase in meat consumption: which percent or value? The P-value?)

●Response: We added the statistical value in the abstract.

(p2 line44,46)

In Methods:

- Meat consumption: the author mean just red meat or is poultry meat included?

●Response: Since we Japanese generally do not distinguish between red meat and poultry (white) meat, "Meat" means both red meat and white meat, and we added that discription.

(p9 line142)

In Results & Discussion:

- Fig 3: The units in the horizontal and vertical axes are not specified. Therefore, this figure cannot be understood on its own.

●Response: We put the units in the horizontal and vertical axes of Fig 3.

- The study relied on self-reported data, which may be subject to recall bias and other inaccuracies.

●Response: We added about recall bias and other inaccuracies in the discussion section.

(p18,19 line304-305,306-309)

- The study did not take into account other potential environmental factors such as smoking, air pollution, and other lifestyle factors.

●Response: We mentioned about other environmental factors in the limitation section.

(p18,19 line306-309)
